# River Surface Velocity and Discharge Estimation Using Optical Flow and Unlabeled Physics-Informed Neural Networks

**DOI:** 10.3390/s26113448

**Published:** 2026-05-29

**Authors:** Zhongyu Shu, Yubo Gao, Guo Zhang, Zihan Xu, Jianping Wang

**Affiliations:** 1 Faculty of Information Engineering and Automation, Kunming University of Science and Technology, Kunming 650500, China; shuzhongyu@stu.kust.edu.cn (Z.S.); zg@kust.edu.cn (G.Z.); xuzihan@stu.kust.edu.cn (Z.X.); 2 School of Metallurgical and Ecological Engineering, University of Science and Technology Beijing, Beijing 100083, China; u202240478@xs.ustb.edu.cn

**Keywords:** river surface velocity, river discharge, physics-informed neural networks, convection–diffusion equation

## Abstract

Quantifying river surface velocity and discharge is essential for flood control and mitigation. Traditional contact measurement methods are capable of providing precise results, yet they demand considerable manpower and material resources and face implementation challenges in flood seasons. Image velocimetry methods have attracted extensive attention due to their low cost, simplicity in operation, and safety. However, most of them lack a physical basis and interpretability. This paper introduces a river flow estimation algorithm combined with Physics-Informed Neural Networks (PINNs). The introduction of the convection–diffusion equation based on optical flow enables the model to better fit the flow characteristics of water and provides stronger physical support for the measurement results. The adoption of this equation as the loss function and the introduction of multiple scenarios eliminate the need for labeled data in the PINNs training process. The experimental results in both artificial and natural river channels demonstrate that the relative errors of the discharge measured by the proposed method are 0.66% and −1.75%, and the relative errors of the mean velocity are 0.64% and −2.33%. Compared with other methods, the proposed method exhibits superior performance.

## 1. Introduction

As one of the key hydrological tasks, the acquisition of river data provides critical decision-making support for flood monitoring and prevention. Real-time, efficient, and accurate measurement of river flow velocity and discharge has become an indispensable means to mitigate the severity of flood disasters. However, natural rivers exhibit complex diversity due to variations in their underlying terrain, and the surrounding natural environment also impedes the implementation of traditional measurement methods. Therefore, exploring efficient and accurate non-contact measurement technologies holds great practical significance [1].

Commonly used image-based non-contact flow velocity and discharge measurement methods include Particle Image Velocimetry (PIV) [2], Large-Scale Particle Image Velocimetry (LSPIV) [3], Space-Time Image Velocimetry (STIV) [4], and the optical flow method, among others. As a traditional non-contact measurement technique, PIV captures flow field information by photographing tracer particles in the target fluid and analyzing their motion trajectories through particle extraction followed by image matching and other procedures. On the basis of PIV, Fujita et al. [3] proposed LSPIV, which utilizes natural floating objects in rivers to replace artificially injected tracer particles, thereby avoiding environmental impacts to a certain extent. However, the application of LSPIV is susceptible to the types and quantities of floating objects in rivers, and it also suffers from poor real-time performance. To address this issue, Fujita et al. [4] put forward STIV. This method sets velocity measurement lines along the flow direction, plots space-time images by recording the variation of grayscale on the measurement lines over time, and calculates flow velocity according to the texture angles in the space-time images. Compared with LSPIV, STIV has a significant improvement in calculation speed. However, this method is highly sensitive to the relative angle between the velocity measurement line and the flow direction, which makes it difficult to achieve high-precision velocity measurement in actual river scenarios with complex flow regimes. The optical flow method breaks free from the limitation of velocity measurement lines. Based on the assumptions of brightness consistency and temporal continuity, it estimates the motion of object images between adjacent frames by leveraging pixel changes and their correlations in sequential images over the time dimension. Horn and Schunck [5] added the global smoothness assumption to the basic hypotheses and proposed the classic Horn-Schunck (H-S) optical flow method. The global smoothness assumption of the H-S method postulates that the optical flow field of the entire image should maintain global smoothness, with abrupt changes in optical flow only permitted at object boundaries. This method computes the optical flow value at each pixel in the image, but it involves complex computations and consumes considerable time. Lucas and Kanade [6] introduced the spatial consistency assumption and proposed the Lucas-Kanade (L-K) optical flow method. The L-K method assumes that pixels within the same local neighborhood of an image have similar motion directions and magnitudes. Nevertheless, in most cases, the target moves rapidly and discontinuously, which violates the small motion constraint and thus introduces substantial errors into the measurements of the L-K method. To remedy this defect, Bouguet [7] proposed the pyramid-based L-K optical flow method, which systematically integrates image pyramids with the affine L-K optical flow method. This integration ensures that fast-moving objects still satisfy the small motion constraint when represented in small-sized images. Farnebäck [8] proposed a method that approximates each neighborhood between adjacent frames using a quadratic polynomial, thereby deriving an approach to estimate the displacement field based on polynomial expansion coefficients. This method not only addresses the accuracy deficiency of traditional methods when dealing with large-displacement and complex motion scenarios but also guarantees favorable stability. In order to solve the problem that current optical flow extraction research mostly focuses on accuracy while neglecting time complexity, which makes it unable to meet the real-time processing requirements in general non-specific field scenarios, Kroeger et al. [9] proposed a fast optical flow calculation method based on Dense Inverse Search (DIS). By adopting inverse search to find block correspondences, multi-scale aggregation to generate dense displacement fields, and variational refinement, this method achieves a significant reduction in time complexity while effectively guaranteeing prediction accuracy.

In recent years, with the rapid development of deep learning technology, the introduction of neural networks has facilitated optical flow analysis under complex conditions. Dosovitskiy et al. [10] proposed FlowNet, the first Convolutional Neural Network (CNN)-based optical flow prediction model, which enables high-precision real-time optical flow estimation through training on synthetic datasets. Ilg et al. [11] put forward FlowNet 2.0. It stacks multiple FlowNetC and FlowNetS, which feature explicit correlation and a simple encoder-decoder architecture respectively, and introduces FlowNetSD, a sub-network specifically optimized for small displacements, and achieves a significant improvement in estimation accuracy. Huang et al. [12] proposed FlowFormer, an optical flow estimation model based on the Transformer architecture, which achieves high-precision and strong generalization in optical flow prediction by constructing a 4D cost volume and performing efficient encoding and decoding. Xu et al. [13] presented the GMFlow model; by abandoning numerous iterative refinement steps and directly modeling pixel correspondences via global matching, while addressing issues such as occlusion and out-of-boundary pixels, this model achieves a balance between large displacement handling capability, accuracy, and efficiency. However, the natural river environment is complex, and the motion states of non-rigid water flow are highly variable [14]. Both traditional methods and deep learning-based optical flow models focus on pixel-level processing and are unable to conduct physical-level analysis of water flow, which means the accuracy of these methods in estimating water flow displacement still needs further improvement.

Due to the proposal of the Universal Approximation Theorem [15], neural networks have been proven to possess the ability to approximate any function. Building on this, Raissi et al. [16] proposed Physics-Informed Neural Networks (PINNs), which incorporate physical prior knowledge into the training of neural networks. They used Fully Connected Neural Networks (FCNNs) to approximate the solutions of partial differential equations (PDEs), including the Schrödinger equation, Allen-Cahn equation, Navier-Stokes equations, and Korteweg-de Vries equation, and obtained promising results. Subsequently, PINNs have been widely applied in various fields. Some studies have proposed novel methods for applying PINNs to fluid velocity measurement. These methods achieve the reconstruction of velocity fields using limited observational data while preserving the physical consistency of the predicted results. Fan et al. [17] proposed a PINN-based pressure field reconstruction method. By embedding the Navier–Stokes equations into the loss function, they demonstrated how to solve for pressure data from velocity fields measured by PIV. Hasanuzzaman et al. [18] proposed a PINN-based data enhancement method for PIV measurements. By embedding the physical constraints of the Reynolds-averaged Navier–Stokes equations, they successfully reconstructed the velocity field of turbulent boundary layers using domain boundary data. Zhang et al. [19] proposed a hybrid framework OF-PhyNet based on optical flow and PINN. This framework extracts the motion characteristics of targets using the H–S optical flow method, and embeds the two-dimensional shallow water equations (SWEs) and the continuity equation into the model to provide physical constraints, thereby achieving robust reconstruction of river surface flow fields. These methods based on PINNs and fluid mechanics knowledge have achieved satisfactory results in the reconstruction of physical fields. However, most of them first obtain measured data from the measurement scenario through traditional approaches and then employ such data for model training, which renders the model performance dependent on data quality to a certain extent. Meanwhile, the specification of such labeled data renders the trained models highly specific to a particular problem or scenario [20]. The necessity of acquiring new data and retraining the model under novel scene conditions further results in limitations in the generalization performance of these methods.

To address the limitations of existing methods, an image-based flow measurement method combining optical flow and PINN is proposed. This method introduces the convection–diffusion equation to provide physical constraints, ensuring that the model predictions conform to fluid laws. Meanwhile, optical flow information derived from multi-scenario images is adopted to replace the measured data of a single scene, which eliminates the dependence of model performance on measured data and overcomes the limitation of generalization ability, thereby realizing label-free measurement of flow velocity and discharge.

## 2. Method

### 2.1. Overview of PINNs

PINNs were originally designed to solve and discover partial differential equations [16]. Consider the following classical form of partial differential equations:

(1) F[u(x,t)]=0,x∈Ω,t∈[0,T]

(2) B[u(x,t)]=0,x∈∂Ω,t∈[0,T]

(3) I[u(x,0)]=0,x∈Ω

In the equations, *x* and *t* represent the spatial coordinates and time coordinates, respectively, while *u* denotes the system state. *F* is a partial differential equation containing several differential operators, *B* stands for the boundary condition (BC), and *I* is the initial condition (IC). This set of equations is capable of describing most physical problems [21], including the wave equation, heat conduction equation, and Poisson equation.

In the classical framework, PINNs are used to solve the target values of specific systems of equations in a designated domain, and the equation solution u(x,t) is approximated by the model output ${\tilde{u}}_{NN}\left(x,t\right)$. To make the model output fit the solution space described by the equations as closely as possible, the loss function of the model consists of two components, whose weights are represented by $\lambda_{f}$ and $\lambda_{u}$, respectively.

(4) $$L=\lambda_{f}\cdot {\mathrm{MSE}}_{f}+\lambda_{u}\cdot {\mathrm{MSE}}_{u}$$

$MSE_{f}$ and $MSE_{u}$ denote the residuals of the partial differential equation and the data fitting loss, respectively, which are given by the following equations:

(5) $${\mathrm{MSE}}_{f}=\frac{1}{N_{f}}\sum_{i=1}^{N_{f}}{\left|F\left[{\tilde{u}}_{NN}\left(x_{f,i},t_{f,i}\right)\right]\right|}^{2}$$

(6) $${\mathrm{MSE}}_{u}=\frac{1}{N_{u}}\sum_{i=1}^{N_{u}}{\left|{\tilde{u}}_{NN}\left(x_{u,i},t_{u,i}\right)-u\left(x_{u,i},t_{u,i}\right)\right|}^{2}$$

In the equations, *N* denotes the number of collocation points, and the subscript i∈[1,n] indicates the *i*-th collocation point. ${\tilde{u}}_{NN}\left(x,t\right)$ represents the output of the neural network. $\left(x_{f},t_{f}\right)$ denotes the collocation points for the partial differential equation, $\left(x_{u},t_{u}\right)$ denotes the collocation points under corresponding data conditions, and $u(x_{u},t_{u})$ denotes the labeled data of the corresponding collocation points.

In the process of equation solving, $\left(x_{u},t_{u}\right)$ can be divided into boundary condition collocation points $\left(x_{B},t_{B}\right)$ and initial condition collocation points $\left(x_{I},t_{I}\right)$, with $\lambda_{B}$ and $\lambda_{I}$ denoting the weights of the losses from these two types of collocation points, respectively. Furthermore, $MSE_{u}$ can be expressed as:

(7) $${\mathrm{MSE}}_{u}=\lambda_{B}\cdot {\mathrm{MSE}}_{B}+\lambda_{I}\cdot {\mathrm{MSE}}_{I}$$

(8) $${\mathrm{MSE}}_{B}=\frac{1}{N_{B}}\sum_{i=1}^{N_{B}}{\left|{\tilde{u}}_{NN}\left(x_{B,i},t_{B,i}\right)-u\left(x_{B,i},t_{B,i}\right)\right|}^{2}$$

(9) $${\mathrm{MSE}}_{I}=\frac{1}{N_{I}}\sum_{i=1}^{N_{I}}{\left|{\tilde{u}}_{NN}\left(x_{I,i},0\right)-u\left(x_{I,i},0\right)\right|}^{2}$$

In the equations, the labeled data at the boundary condition collocation points and initial condition collocation points are denoted by $\left(x_{B,i},t_{B,i}\right)$ and $\left(x_{I,i},0\right)$, respectively, and the corresponding model outputs are denoted by ${\tilde{u}}_{NN}\left(x_{B,i},t_{B,i}\right)$ and ${\tilde{u}}_{NN}\left(x_{I,i},0\right)$, respectively.

For equation discovery, its purpose is to infer the parameters of the partial differential equation for fitting through observed data. Therefore, $\left(x_{u},t_{u}\right)$ denotes the observation collocation points $\left(x_{O},t_{O}\right)$ for data acquisition, and $u(x_{u},t_{u})$ denotes the data $u(x_{O},t_{O})$ obtained from the corresponding observation points. Furthermore, $MSE_{u}$ can be expressed as:

(10) $${\mathrm{MSE}}_{u}=\frac{1}{N}\sum_{i=1}^{N}{\left|{\tilde{u}}_{NN}\left(x_{O,i},t_{O,i}\right)-u\left(x_{O,i},t_{O,i}\right)\right|}^{2}$$

Starting from randomly initialized parameters, the model receives inputs for forward propagation to compute predicted values and the initial loss. Subsequently, backpropagation is performed to calculate the gradients of the loss with respect to the model’s learnable parameters based on automatic differentiation. The gradient descent method is used to adjust the parameters in the direction of decreasing loss to achieve residual minimization. Through iterative training, the model can ultimately approximate the state space described by the system of partial differential equations.

### 2.2. Convection–Diffusion Equation

As an important branch of partial differential equations, the convection–diffusion equation describes the diffusive motion of a certain physical quantity (such as concentration, temperature, etc.) in a fluid under transport, and is widely used in the field of fluid mechanics. In a two-dimensional field, the convection–diffusion equation without sources and chemical reactions has the following form:

(11) $$\frac{\partial c}{\partial t}+\nabla \cdot \left(\vec{u}c\right)=D\nabla^{2}c$$

In the equation, *c* denotes the transported scalar field, $\vec{u}=u_{x}\vec{i}+v_{y}\vec{j}$ is the velocity vector, *D* is the diffusion coefficient of *c*, $\nabla =\frac{\partial }{\partial x}\vec{i}+\frac{\partial }{\partial y}\vec{j}$ is the vector differential operator, and $\nabla^{2}=\frac{\partial^{2}}{\partial x^{2}}+\frac{\partial^{2}}{\partial y^{2}}$ is the Laplace operator.

As one of the basic conservation equations in fluid mechanics, the continuity equation describes the law of mass conservation for fluids in motion. In its two-dimensional form, the continuity equation is expressed as follows:

(12) $$\frac{\partial \rho }{\partial t}+\nabla \cdot \left(\vec{u}\rho \right)=0$$

In the equation, ρ denotes the fluid density.

In general, it is usually assumed that the fluid is incompressible, and thus ρ is a constant. In this case, ρ can be factored out of the divergence operation, yielding the following result:

(13) $$\frac{\partial \rho }{\partial t}=0$$

At this point, the continuity equation can be transformed into:

(14) $$\rho \cdot \nabla \vec{u}=0$$

Since ρ is not zero, the following result can be obtained:

(15) $$\nabla \cdot \vec{u}=0$$

Substituting Equation (15) into Equation (11), the following result can be obtained:

(16) $$\frac{\partial c}{\partial t}+\vec{u}\cdot \nabla c=D\nabla^{2}c$$

Expanding it, the two-dimensional convection–diffusion equation for incompressible fluids without sources and chemical reactions can be obtained:

(17) $$\frac{\partial c}{\partial t}+u_{x}\cdot \frac{\partial c}{\partial x}+v_{y}\cdot \frac{\partial c}{\partial y}=D\left(\frac{\partial^{2}c}{\partial x^{2}}+\frac{\partial^{2}c}{\partial y^{2}}\right)$$

For the convection–diffusion equation, the forward problem is to determine the scalar concentration field given the velocity field, initial conditions, and boundary conditions. Therefore, solving the inverse problem of the equation allows for the determination of the velocity field given the corresponding scalar concentration field.

### 2.3. Improvement of Model Generalization

Different from forward problems whose outputs are usually unique and deterministic, inverse problems infer the system inputs, internal parameters or structural characteristics that lead to such outputs from the known system outputs or observation data, and thus generally exhibit Hadamard ill-posedness.

In classical PINN frameworks, this ill-posedness is conventionally addressed by a composite loss function comprising the PDE residual $MSE_{f}$ and a data-fitting term $MSE_{u}$, where $MSE_{u}$ ensures that the model predictions conform to prescribed boundary conditions and initial conditions. In practical measurements, these boundary and initial conditions are replaced by observational data [19].

Current research predominantly acquires data through conventional methods and employs it as $MSE_{u}$ for model training. However, this practice restricts the prediction accuracy of the model to the measurement data obtained from conventional algorithms. Furthermore, labeled data collected under specific scenarios confine the model to settings consistent with the observations, depriving the network of generalizability across different scenarios and necessitating retraining when handling different conditions [22].

To address this issue, the proposed method omits the explicit data-fitting loss $MSE_{u}$, thereby relieving the model from data-fitting constraints and allowing its outputs access to an open solution space. Meanwhile, to enable the model to perceive environmental changes, its inputs are adjusted to image grayscale gradients instead of the spatiotemporal coordinates used in standard PINNs. To re-stabilize the solution, multi-scenario, unlabeled optical-flow gradient data are incorporated into the model training process.

Previous studies have shown that neural networks inherently tend to learn low-frequency and smooth functions during training [23], which provides certain assistance for model convergence. Except for localized turbulence near obstacles, the velocity field is predominantly smooth and continuous at the spatial scales captured by riverbank cameras. This training tendency acts as an implicit regularizer, penalizing high-frequency components and suppressing non-physical oscillatory solutions in the inferred velocity field, thereby contributing to the stability of the derived results.

For a single river scene, the PDE residual alone admits infinitely many velocity fields. However, across different rivers with varying environmental conditions, the underlying physical laws remain invariant. Consequently, their corresponding sets of admissible solutions heuristically tend to intersect in a region that contracts progressively as scenario diversity increases. By exposing the network to gray-gradient arrays extracted from diverse river channels during training, the model is compelled to learn a generalizable mapping from brightness transport features to velocity vectors that holds universally across scenarios. Each additional training scenario functions as an independent physical realization that prunes away non-generalizable branches of the solution space; this cross-scene training objective implicitly penalizes overfitting to scenario-specific observational biases, as such memorization would produce scene-specific velocity fields that violate the underlying physics when applied to other training rivers, thereby increasing the overall PDE residual loss across the training set. In this paradigm, the diversity of training data acts as an implicit regularizer that distributes physical constraints throughout the learned parameter space, progressively narrowing the volume of admissible solutions without requiring labeled observational data for model training. The model thereby acquires the capacity to adapt to previously unseen river scenes by extracting the relationship between optical-flow gradients and surface velocity, effectively substituting cross-scene empirical constraints for conventional observational data that are typically indispensable in standard PINN frameworks.

### 2.4. Loss Function

Corpetti et al. [24] proposed that the image brightness is approximately proportional to the vertical integral of mass density when processing cloud images, i.e., I∝∫cdz. For 2D images, the depth *z* is constant, thus yielding I∝c. Subsequent studies [25,26] have demonstrated the reasonableness of introducing this assumption into natural river environments. Hence, Equation (17) can be transformed and expanded into:

(18) $$\frac{\partial I}{\partial t}+u_{x}\frac{\partial I}{\partial x}+v_{y}\frac{\partial I}{\partial y}=D\left(\frac{\partial^{2}I}{\partial x^{2}}+\frac{\partial^{2}I}{\partial y^{2}}\right)$$

In the equation, $\frac{\partial I}{\partial x}$, $\frac{\partial I}{\partial y}$ and $\frac{\partial I}{\partial t}$ denote the first-order partial derivatives of the grayscale *I* in the *x*, *y* and *t* directions, respectively, and $\frac{\partial^{2}I}{\partial x^{2}}$ and $\frac{\partial^{2}I}{\partial y^{2}}$ denote the second-order partial derivatives of the grayscale *I* in the *x* and *y* directions, respectively. $\frac{\partial I}{\partial x}$, $\frac{\partial I}{\partial y}$, $\frac{\partial I}{\partial t}$, $\frac{\partial^{2}I}{\partial x^{2}}$ and $\frac{\partial^{2}I}{\partial y^{2}}$ are denoted as $I_{x}$, $I_{y}$, $I_{t}$, $I_{xx}$ and $I_{yy}$, which are calculated using the following difference schemes:

(19) $$I_{x}=\frac{I_{1}\left(x+1,y\right)-I_{1}\left(x-1,y\right)}{2}$$

(20) $$I_{y}=\frac{I_{1}\left(x,y+1\right)-I_{1}\left(x,y-1\right)}{2}$$

(21) $$I_{t}=I_{2}\left(x,y\right)-I_{1}\left(x,y\right)$$

(22) $$I_{xx}=I_{1}\left(x+1,y\right)-2I_{1}\left(x,y\right)+I_{1}\left(x-1,y\right)$$

(23) $$I_{yy}=I_{1}\left(x,y+1\right)-2I_{1}\left(x,y\right)+I_{1}\left(x,y-1\right)$$

In the neural network model proposed in this paper, the input X is set as a five-dimensional array representing the five partial derivatives for calculation, i.e., $X=\left[I_{x},I_{y},I_{t},I_{xx},I_{yy}\right]$. The model is designed to output a two-dimensional array representing the velocities in the *x* and *y* directions of the computational domain, i.e., $\left[u_{x},v_{y}\right]$.

The predictive results of the model depend on the pixel grayscale values and their gradients, which are affected by illumination. However, in natural river environments, variations in light intensity are far more drastic than in indoor settings, leading to severe fluctuations in image grayscale values and a significantly higher likelihood of outliers in such regions. In this scenario, a loss function formulated as Mean Squared Error (MSE) renders the training process more susceptible to being dominated by outliers, causing the model to tend toward fitting a small number of anomalous points rather than adhering to the underlying physical laws. To enable the model to handle such conditions robustly, the loss function of the proposed model adopts the form of Mean Absolute Error (MAE), since MAE exhibits stronger robustness against errors induced by lighting conditions as well as outlier anomalies [27]. Therefore, the model is trained by minimizing the following physics-based loss function:

(24) $$L=\frac{1}{N}\sum_{i=1}^{N}\left|I_{t,i}+u_{x,i}I_{x,i}+v_{y,i}I_{y,i}-D\left(I_{xx,i}+I_{yy,i}\right)\right|$$

In the equation, the subscript $i\in \left[1,N\right]$ denotes the *i*-th array, and *N* denotes the total number of arrays.

### 2.5. Network Model Design

**Figure 1:**
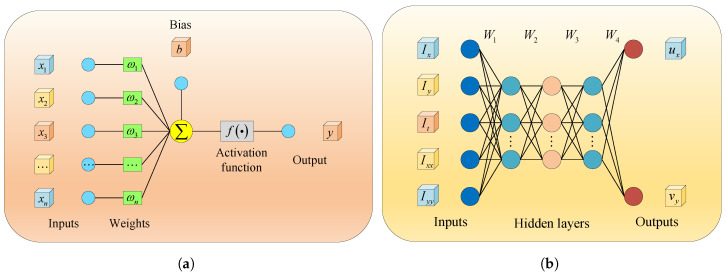
(**a**) Schematic diagram of the neuron. Blue circles denote data interfaces for inputs, bias term, and output. Alternating colors improve visual distinction of adjacent channels. (**b**) Schematic diagram of the MLP architecture. The labels $W_{i}$ represent the weight matrices connecting adjacent layers. Alternating colors distinguish adjacent inputs, hidden layers, and outputs, respectively.

Multilayer Perceptrons (MLPs) are one of the most widely used types of neural networks [28]. They are capable of learning high-order feature representations from data through multi-layer nonlinear transformations, and their basic units consist of neurons. As shown in Figure 1a, for the output signals $X=[x_{1},x_{2},\ldots ,x_{n}]$ from *n* different neurons in the previous layer, the neuron receives them through corresponding input data interfaces, performs a weighted summation of these signals with respective weights, introduces a bias term through a bias data interface, and then processes the aggregated result through an activation function. Finally, the neuron transmits the result through an output data interface. The model can be expressed as:

(25) $$y=f\left(\sum_{i=1}^{n}\omega_{i}x_{i}+b\right)$$

In the Equation (25), $x_{i}$ denotes the input term, $\omega_{i}$ denotes the weight, *b* denotes the bias term, *f* denotes the activation function, and *y* denotes the output term.

Compared with single-layer perceptrons, MLPs perform better in fitting complex functions. When an MLP contains a sufficient number of hidden-layer neurons, it can approximate any complex nonlinear function with arbitrary precision. An MLP usually consists of an input layer, one or more hidden layers, and an output layer, with neurons in each layer connected by weights and biases. For an input x, the *l*-th hidden layer has a hidden variable $y^{\left(l\right)}$. And the L-layer deep neural network can be expressed as:

(26) $$\left\{\begin{array}{c} y^{\left(l\right)}=x,\;l=0\\ y^{\left(l\right)}=f\left(W^{\left(l\right)}y^{\left(l-1\right)}+b^{\left(l\right)}\right),\;1\leq l\leq L-1\\ y^{\left(l\right)}=W^{\left(l\right)}y^{\left(l-1\right)}+b^{\left(l\right)},\;l=L \end{array}\right.$$

In the formula, $W^{\left(l\right)}$ and $b^{\left(l\right)}$ denote the weight matrix and bias of the *l*-th neural network layer, respectively, which are the trainable parameters of the neural network [29].

Figure 1b shows the MLP architecture adopted in this paper. In the process of architecture selection, this study prioritizes the overall accuracy of predictions, supplemented by the correlation between predicted results and ground truth. In iterative experiments, the baseline MLP model consists of one hidden layer with 100 neurons. The adjustment priority between network depth and the number of neurons per layer is first determined through preliminary testing; based on this, the network depth and the number of neurons per layer are sequentially adjusted to gradually increase model complexity and approach better results. The final MLP employed in this study comprises three hidden layers, each with 1000 neurons, capturing the coupling relationship between optical flow gradients and flow velocity through sufficient parameter space, thereby fitting the nonlinear fluid characteristics represented by the advection-diffusion equation. Due to its low computational cost and ability to effectively alleviate the vanishing gradient problem [30], the ReLU function is selected as the baseline activation function for testing in this paper. In subsequent tests, it is evaluated alongside Sigmoid and Tanh, which are also widely used in neural network applications. The final activation function is determined after systematic testing and comparison.

To date, there is no unified theory to guide the design of an appropriate neural network [31]. Under suitable conditions, increasing the complexity of a neural network can exert a positive effect on model performance, yet this effect is not linear. When the model complexity exceeds a certain threshold, the performance gains will diminish [32]. Therefore, the goal of network design shifts to maintaining as low a complexity and computational cost as possible while ensuring the network achieves the desired accuracy. This principle generally helps to develop artificial intelligence models with fast learning speeds and excellent predictive ability, while avoiding the problem of overfitting. It should be noted, however, that the network used in this paper is not the optimal solution to this problem, but rather the best choice obtained through the testing process.

### 2.6. Model Training

The Adam optimizer is used to minimize the residual loss constituted by partial differential equations. Based on the first-order and second-order moments of gradients, the Adam optimizer calculates an adaptive update step size for each parameter, which addresses the issues of difficult learning rate adjustment and slow convergence in traditional Stochastic Gradient Descent (SGD). During the training process, the model output and corresponding residual loss are first calculated through forward propagation. Then, the gradients are cleared, and automatic differentiation technology is used to compute the gradients of the residual loss with respect to the model parameters. Subsequently, the Adam optimizer calculates an adaptive step size based on the gradients and moments, and updates the model parameters to minimize the residual loss. Both the initial learning rate and the maximum number of training epochs are determined through comparative testing: starting from the smallest candidate values, they are progressively increased, and the final selection is made based on the corresponding model prediction performance. Through this systematic comparison, the optimal configuration was identified as an initial learning rate of $5\times 10^{-4}$ and a maximum of 7500 training epochs.

**Figure 2:**
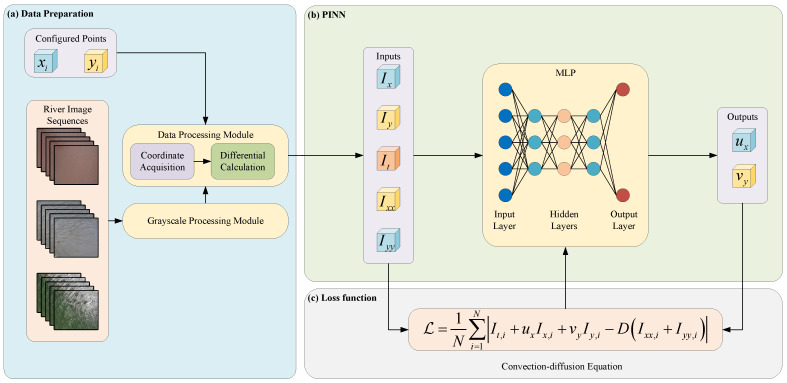
Schematic diagram of the PINN. The framework consists of three main modules. Different modules are distinguished by different colors, and arrows indicate the data flow. (**a**) Data preparation module, which converts raw river image sequences into grayscale images, acquires configured point data, and extracts optical flow gradient data required by the model based on the configured point coordinates. (**b**) PINN module, where the network architecture is composed of an MLP that receives optical flow gradient data and outputs corresponding velocity fields. (**c**) Loss function module, which receives model inputs and outputs, constructs a physics-constrained loss function based on the convection–diffusion equation, and optimizes the network parameters by minimizing this loss function during training.

Figure 2 illustrates the schematic diagram of the PINN framework proposed in this paper for solving river surface flow velocities by combining the convection–diffusion equation. To ensure sufficient diversity of the data and enable the model to fit flow characteristics under different conditions, multiple sets of continuous frame images of rivers are adopted. This multi-scenario training strategy not only improves the model’s adaptability to diverse hydraulic conditions but also serves as an implicit regularization mechanism. Specifically, by learning from diverse illumination environments, the model becomes less sensitive to scene-specific noise and avoids overfitting to local outliers in individual scenarios. Meanwhile, the collocation points used for training can be randomly selected on the images, and the number of collocation points can also be specified arbitrarily. The model training steps are as follows:

1. For a series of continuous frame images of rivers used for training, the collocation points and their corresponding grayscale data are obtained and preprocessed into input arrays;
2. Define the network architecture;
3. Initialize the network parameters;
4. Compute the outputs via the neural network;
5. Compute the loss function based on the inputs and outputs;
6. Update the neural network parameters;
7. Repeat Steps 4 to 6 until the specified number of iterations is reached.

### 2.7. Projection Transformation

To determine the mapping relationship between real-world coordinates and pixel coordinates, at least four horizontally coplanar calibration points are arranged on both banks of the river. A coordinate system is established with one of the calibration points as the origin, and the real-world coordinates of all calibration points are determined. Meanwhile, the corresponding pixel coordinates of these calibration points can be acquired from the captured images.

By correlating the coordinates of calibration points in different coordinate systems, the projection transformation matrix $p_{1}=\left[\begin{array}{ccc} \lambda_{11} & \lambda_{12} & \lambda_{13}\\ \lambda_{21} & \lambda_{22} & \lambda_{23}\\ \lambda_{31} & \lambda_{32} & \lambda_{33} \end{array}\right]$ can be derived based on the principle of projective transformation. The correspondence between real-world coordinates $\left(x,y,1\right)$ and captured image coordinates $\left(a,b\right)$ can then be expressed as:

(27) $$\left[\begin{array}{c} x^{\prime }\\ y^{\prime }\\ z^{\prime } \end{array}\right]=\left[\begin{array}{ccc} \lambda_{11} & \lambda_{12} & \lambda_{13}\\ \lambda_{21} & \lambda_{22} & \lambda_{23}\\ \lambda_{31} & \lambda_{32} & \lambda_{33} \end{array}\right]\left[\begin{array}{c} a\\ b\\ 1 \end{array}\right]$$

(28) $$\left\{\begin{array}{c} x^{\prime }=\lambda_{11}a+\lambda_{12}b+\lambda_{13}\\ y^{\prime }=\lambda_{21}a+\lambda_{22}b+\lambda_{23}\\ z^{\prime }=\lambda_{31}a+\lambda_{32}b+\lambda_{33} \end{array}\right.$$

(29) $$\left\{\begin{array}{c} x=\frac{x^{\prime }}{z^{\prime }}\\ y=\frac{y^{\prime }}{z^{\prime }} \end{array}\right.$$

According to the projection transformation matrix $p_{1}$ and the real-world coordinates of the velocity measurement points, the pixel coordinates of these points can be obtained.

Meanwhile, river images captured by cameras show different characteristics due to factors such as camera installation height and actual shooting angle. To ensure the prediction accuracy of the model, the projective transformation principle is used to convert the captured view to a bird’s-eye view, so that the flow velocity calculation can be carried out under a unified standard.

A region containing the actual velocity measurement points is selected on the river image, and the size and coordinates of the bird’s-eye view are defined. The selected region can then be converted into a bird’s-eye view using projective transformation, and the coordinates of the velocity measurement points in the bird’s-eye view can be calculated via the corresponding transformation matrix.

For a series of bird’s-eye view images obtained by processing the images of the river to be measured, the partial derivative array $\left[I_{x},I_{y},I_{t},I_{xx},I_{yy}\right]$ at the coordinates of the velocity measurement point is calculated frame by frame based on the coordinates of the velocity measurement point in the bird’s-eye view. This array is input into the trained model to obtain the frame-by-frame velocity array at the velocity measurement point in the bird’s-eye view, and the frame-by-frame average velocity $\left[u_{x},v_{y}\right]$ is derived by calculating the mean value. The transformation relationships among the bird’s-eye view image coordinate system, the captured image coordinate system, and the real-world coordinate system can be obtained via the projective transformation matrix, further yielding the velocity array $\left[u_{r},v_{r}\right]$ of the velocity measurement point in the real-world coordinates. The magnitude of this array is calculated and divided by the time interval between two adjacent frames, thereby enabling the calculation of the actual surface flow velocity of the river at the corresponding velocity measurement point.

### 2.8. Calculation of Total Discharge and Mean Flow Velocity

**Figure 3:**
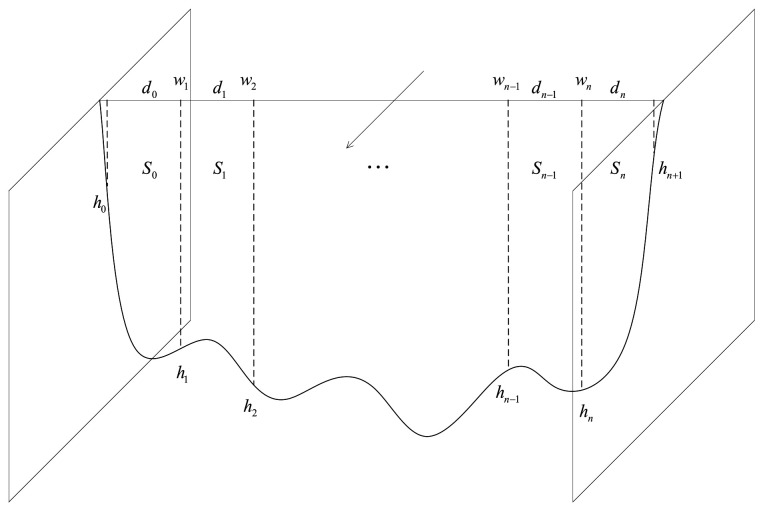
Schematic diagram of the velocity-area method. The arrow denotes river flow direction, and the dashed lines illustrate the arrangement of velocity measurement points.

After obtaining the surface flow velocities at all velocity measurement points, the total discharge and mean flow velocity of the river can be calculated using the velocity-area method.As shown in Figure 3, let the velocity at the *i*-th measurement point be $w_{i}\left(i=1,2,...,n\right)$, and *n* be the number of measurement points.The distance between the *i*-th and the (i+1)-th measurement points is $d_{i}$, and the distances from the two end measurement points to the river banks are $d_{0}$ and $d_{n}$, respectively.The water depth at the *i*-th measurement point is $h_{i}$, and the water depths at the two river banks are $h_{0}$ and $h_{n+1}$, respectively.

The cross-sectional area $S_{i}$ between a velocity measurement point and the river bank, as well as between the *i*-th and (i+1)-th velocity measurement points, can be approximated by Equation (30):

(30) $$S_{i}=\frac{h_{i}+h_{i+1}}{2}\cdot d_{i}\left(i=0,1,\ldots ,n\right)$$

The partial surface flow velocity corresponding to the cross-section $S_{i}$ is:

(31) $$\left\{\begin{array}{c} \bar{w_{0}}=w_{1}\cdot \mu_{1}\\ \bar{w_{i}}=\frac{w_{i}+w_{i+1}}{2},\;\left(i=1,2,\ldots ,n-1\right)\\ \bar{w_{n}}=w_{n}\cdot \mu_{2} \end{array}\right.$$

In the formula, $\mu_{1}$ and $\mu_{2}$ are the bank coefficients of the two river banks, respectively.

The partial vertical flow velocity can be obtained from the partial surface flow velocity:

(32) $$w_{i}^{\prime }=\bar{w_{i}}\cdot k\;\left(i=0,1,\ldots ,n\right)$$

In the formula, *k* is the surface velocity coefficient, which is provided by the hydrological station.

The total river discharge can be obtained using the velocity-area method:

(33) $$Q=\sum_{i=0}^{n}w_{i}^{\prime }\cdot S_{i}$$

The mean flow velocity of the river can be obtained from the total discharge and the cross-sectional area:

(34) $$\bar{w}=\frac{Q}{\sum_{i=0}^{n}S_{i}}$$

## 3. Experiments and Evaluation Indicators

This section presents two case studies for testing the network proposed in this paper. It should be noted that, to assess the model’s adaptability across different scenarios, the optical flow gradient data used for training were collected from multiple independent river environments, while the cases were excluded from these training sources. Case 1 adopts the Liancheng Hydrological Station in Dali City, Yunnan Province as the experimental site, serving as the validation scenario for architecture selection and hyperparameter tuning. The second experiment uses the captured data from the Gaoqiao Hydrological Station in Chuxiong City, Yunnan Province, which constitutes a held-out test scenario to evaluate the model’s performance in a different river environment. Meanwhile, Case 1 and Case 2 respectively compare and evaluate the estimation performance of the new method and existing image-based flow measurement methods in the artificial river and the natural river.

### 3.1. Experiments

#### 3.1.1. Artificial River Scenario in Dali City

**Figure 4:**
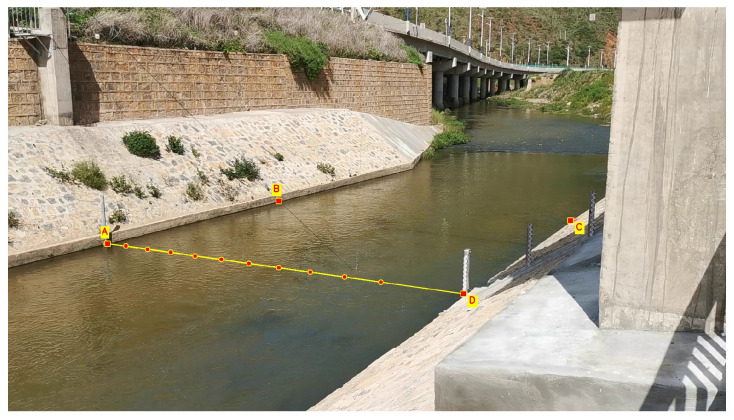
Captured image of Dali Hydrological Station. Four control points are marked A, B, C and D. The line between A and B acts as the flow velocity measurement line, with circles indicating the layout of velocity measurement points along it.

The river at the Liancheng Station in Dali City is an artificial river channel. Based on the years of measured experience at this hydrological station, the left and right bank coefficients of the river are 0.8, the surface velocity coefficient is 0.82, and the channel width is 11.9 m. In general, relatively smooth artificial river channels are characterized by regular shape and low roughness, which can ensure stable flow conditions. Figure 4 shows the captured images in this scenario. Through frame extraction processing of the captured video, 325 frame images are obtained.

Four control points A, B, C, and D are selected on both sides of the river channel. Among them, the line connecting A and D serves as both the river cross-section line and the velocity measurement line. Taking point D as the starting point, measurements are conducted every 1 m within the distance range of 2–11 m from point D using a current meter, which are taken as the vertical average flow velocity at the corresponding velocity measurement points.

#### 3.1.2. Natural River Scenario in Chuxiong City

**Figure 5:**
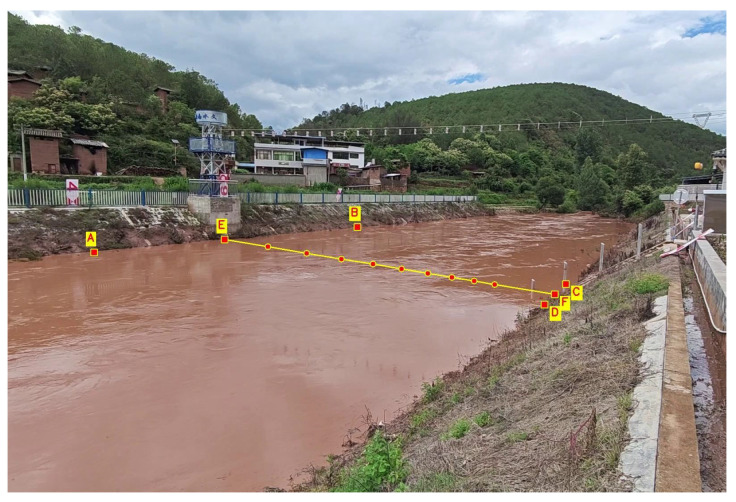
Captured image of Chuxiong Hydrological Station. Four control points are marked A, B, C and D. The line between the other two marked points E and F acts as the flow velocity measurement line, with circles indicating the layout of velocity measurement points along it.

The river at Gaoqiao Station in Chuxiong City is a natural channel with irregular riverbanks and significant variations in cross-sectional flow velocity. Based on the measurement experience of the hydrological station, the left and right bank coefficients of this river are 0.8 and 0.7, respectively, the surface velocity coefficient is 0.97, and the channel width is 24.9 m. During the measurement period, the river was filmed, and 351 frame images were obtained after frame extraction and screening. Constrained by actual shooting conditions, the images captured in this scenario suffer from severe perspective distortion, which further validates the applicability of the proposed method to such challenging scenes. Figure 5 shows the captured footage of this scene.

Four control points A, B, C, and D are selected on both sides of the river channel. The line connecting E and F serves as both the river cross-section line and the velocity measurement line. Taking point F as the starting point, measurements are conducted every 2 m within the distance range of 6.9–22.9 m from point F using a current meter, which are taken as the vertical average flow velocity at the corresponding velocity measurement points.

### 3.2. Evaluation Indicators

In current river flow measurements, although the results obtained by current meters contain some margin of error, these errors are relatively small. Therefore, the results from current meters are generally regarded as the corresponding reference values. The rotor current meter used in the experiments of this study complies with the Chinese national standard GB/T 11826-2019 “Rotating-element current-meters” [33], and its measurement uncertainty is within a controllable range. Meanwhile, the distribution of sampling points as well as the data collection process also conforms to the relevant specifications, which ensures that the collected data can adequately represent the velocity distribution across the entire river cross-section. Consequently, the measurement results are acceptable. Thus, in the comparative tests conducted, the measurement results from the current meter are used as the ground truth. The evaluation of model performance is based on array $\alpha =[\alpha_{1},\alpha_{2},\ldots ,\alpha_{n}]$ obtained from the current meter measurements, array $\beta =[\beta_{1},\beta_{2},\ldots ,\beta_{n}]$ obtained from different image-based flow measurement methods at *n* velocity measurement points, as well as the total cross-sectional discharge and mean flow velocity calculated by these methods.

This paper adopts the following indicators to evaluate model performance. The first one is the root mean square error (RMSE) of the measured data from each method. RMSE quantifies the deviation between different methods and the ground truth to assess their overall accuracy, which is calculated by Equation (35):

(35) $$\mathrm{RMSE}=\sqrt{\frac{1}{n}\sum_{i=1}^{n}{\left(\alpha_{i}-\beta_{i}\right)}^{2}}$$

For individual velocity measurement points, relative error is used to quantify the measurement accuracy and characterize the error of local measurement results. Absolute error represents the difference between the measured values of different methods and the ground truth, while relative error is calculated as the ratio of absolute error to the ground truth and expressed in percentage form. Relative error eliminates the influence of dimensions and numerical magnitudes, and can better reflect the deviation degree between measured values and true values. Meanwhile, relative error is also applied to evaluate the measurement accuracy of total cross-sectional discharge and mean flow velocity for different methods.

Furthermore, the standard deviation (SD) of absolute measurement error is introduced to evaluate the dispersion degree of the measurement error distribution. The SD value quantitatively reflects the fluctuation and stability of the measurement results, which is used to characterize the measurement uncertainty and reliability.

Finally, the Pearson correlation coefficient is used to measure the strength and direction of the linear relationship between the measured values of different methods and the ground truth, so as to assess their consistency. The Pearson correlation coefficient *r* is calculated by Equation (36):

(36) $$r=\frac{\sum_{i=1}^{n}\left(\alpha_{i}-\bar{\alpha }\right)\left(\beta_{i}-\bar{\beta }\right)}{\sqrt{\sum_{i=1}^{n}{\left(\alpha_{i}-\bar{\alpha }\right)}^{2}}\sqrt{\sum_{i=1}^{n}{\left(\beta_{i}-\bar{\beta }\right)}^{2}}}$$

## 4. Results and Discussion

### 4.1. Results of Case Study 1

#### 4.1.1. Analysis of Network Model Structure

The network structure adopted in this paper is obtained through repeated tests. On the premise of ensuring prediction accuracy, the model complexity is kept as low as possible. Therefore, the testing strategy is to start with the simplest network and gradually increase the network complexity until the prediction results meet the target accuracy.

In the test experiments, the numbers of hidden layers and neurons of the MLP start from 1 and 100, respectively. Priority is given to the variation of the overall prediction accuracy (RMSE) of the neural network with its architecture. The models are named P-X-Y, where X denotes the number of hidden layers of the model, and Y denotes the number of neurons contained in a single hidden layer of the model.

**Figure 6:**
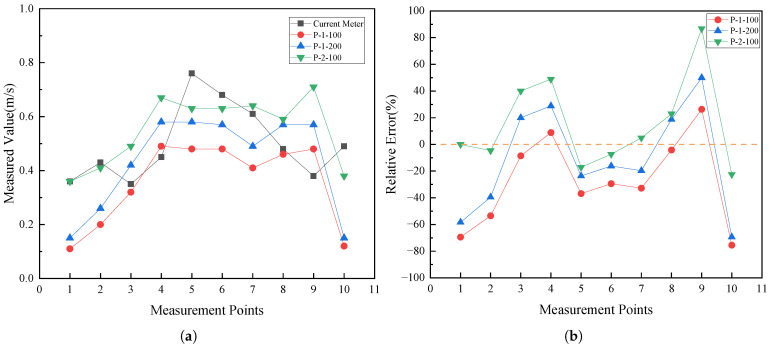
Results of initial tests. (**a**) Measured values of different models; (**b**) relative errors of different models, with the zero-error line represented by a dashed line.

Figure 6 presents the measured values obtained by the current meter, the initial model, the model with two hidden layers and the model with 200 neurons in a single hidden layer, as well as the relative measurement errors of the three models with respect to the current meter. As shown in Figure 6, when the initial model is used to predict the flow velocity, its measured values are lower than those of the current meter at most measurement points. After increasing the number of model parameters, the flow velocity measurements of the two more complex models exhibit an overall upward trend. Although this leads to increased errors at certain measurement points, the complex models produce smaller measurement errors than the initial model at most measurement points.

The measurement accuracies of the three models involved in the preliminary experiments are listed in Table 1. The results show that increasing the model complexity exerts a positive effect on the prediction accuracy of the network model in the preliminary tests. The flow velocity measurement curves of the two more complex models fit much more closely with the measurement line of the current meter, and their overall errors are smaller. On this basis, the measurement accuracy of model P-2-100 is superior to that of P-1-200, which indicates that increasing the number of hidden layers yields a more pronounced improvement in the model measurement accuracy than increasing the number of neurons in a single hidden layer.

Although a network with a single hidden layer is sufficient to effectively represent any function, such a network architecture must be sufficiently large. In many cases, increasing the number of network layers can effectively reduce the number of parameters required by the model [34]. Therefore, on the premise of maintaining as low a model complexity as possible, subsequent test experiments will prioritize increasing the number of hidden layers of the model, and then increase the number of neurons in a single hidden layer on this basis.

**Figure 7:**
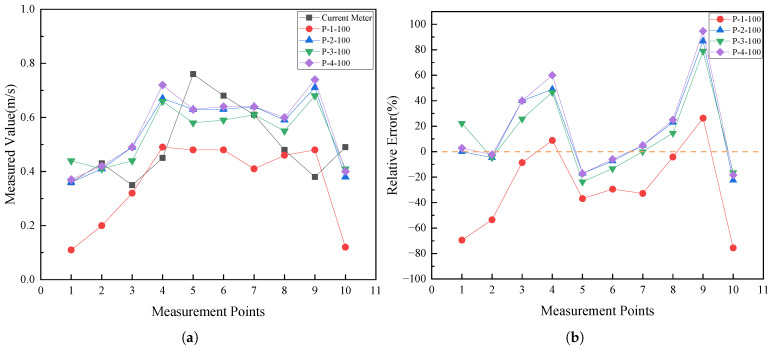
Results of network layer-number tests. (**a**) Measured values of different models; (**b**) relative errors of different models, with the zero-error line represented by a dashed line.

Figure 7 shows the comparison between the prediction results of models with gradually increasing number of hidden layers based on the initial model and the measured values of the current meter, so as to explore the appropriate selection of the number of hidden layers. It can be seen from Figure 7 that compared with the initial model containing only one hidden layer, the measurement curves of the other models are closer to the measurement curve of the current meter. Meanwhile, their measurement results fluctuate correspondingly with the change in the number of layers.

Table 2 summarizes the prediction accuracies of the different neural network models used in the layer number test. Analysis of the data in Table 2 shows that, starting from the initial model, the prediction accuracy of the model gradually increases as the number of hidden layers increases, which indicates that this operation can improve the model performance. However, when the number of hidden layers reaches 4, the model prediction accuracy decreases, and its measurement accuracy is even lower than that of the test model with only 2 hidden layers. This may be related to overfitting caused by an excessively deep network. Therefore, subsequent experiments will be carried out on the model with 3 hidden layers, and the prediction performance of the model will be further improved by increasing the number of neurons in a single layer.

**Figure 8:**
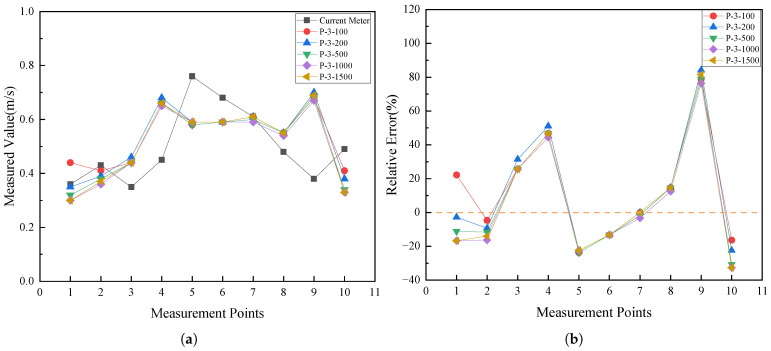
Results of neuron-number tests. (**a**) Measured values of different models; (**b**) relative errors of different models, with the zero-error line represented by a dashed line.

Figure 8 presents a comparison between the prediction results obtained by gradually increasing the number of neurons in a single layer of the network model based on the three-hidden-layer model and the measured values of the current meter. By keeping the number of hidden layers unchanged, different numbers of neurons are tested to explore the most suitable model parameters.

The prediction accuracies of different network models in the neuron number test are shown in Table 3. The data indicate that the performance of different models varies slightly under the condition of three hidden layers. This is because the flow in the central region of the river is stable and the prediction difficulty is low. For most velocity measurement points in the middle of the river, only a small number of parameters are required to achieve stable prediction results. Therefore, the prediction results of different models are relatively close in this region, which further keeps the overall prediction accuracy of the models stable. However, in the regions near the two banks, models with different parameters exhibit large prediction differences. Therefore, on this basis, the Pearson correlation coefficient is introduced to determine the optimal selection of model parameters.

When the number of neurons is less than 1000, the correlation between the model predicted values and the current meter measurements gradually increases with the increase in the number of neurons. However, when the number of neurons exceeds 1000, the correlation decreases instead. Meanwhile, the increase in the number of parameters significantly prolongs the model training time. Therefore, the final parameters of the model are determined as 3 hidden layers with 1000 neurons in each hidden layer.

#### 4.1.2. Analysis of Model Configuration

This section illustrates the heuristic approach followed to determine the best possible design of the model configuration, encompassing the activation function, initial learning rate, and training epochs. The model structure selected for testing is P-3-1000.

**Figure 9:**
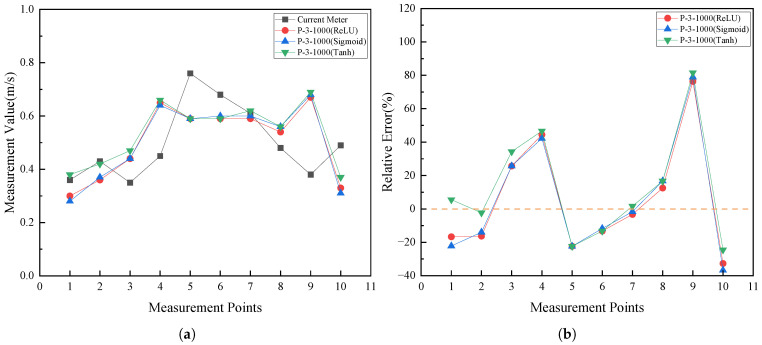
Results of activation function tests. (**a**) Measured values of different models; (**b**) relative errors of different models, with the zero-error line represented by a dashed line.

To determine the optimal activation function, we evaluated three commonly adopted candidates: ReLU, Sigmoid, and Tanh. The measurement results for models equipped with these activation functions are presented in Figure 9, with the corresponding performance metrics summarized in Table 4.

For the P-3-1000 architecture, the Sigmoid-based model yielded a higher RMSE, whereas the ReLU-equipped model attained the optimal performance. Regarding the Pearson correlation coefficient, the ReLU-based model was marginally lower than that of Sigmoid yet still superior to the Tanh-based counterpart. Consequently, ReLU was adopted as the activation function for the proposed model.

**Figure 10:**
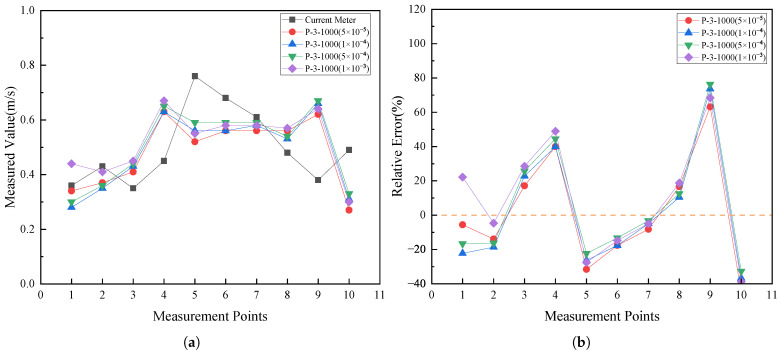
Results of learning rate tests. (**a**) Measured values of different models; (**b**) relative errors of different models, with the zero-error line represented by a dashed line.

Although the Adam optimizer is capable of adaptively adjusting the learning rate for each parameter, the selection of the initial learning rate remains critically important. An excessively small initial learning rate may cause the model to stagnate at saddle points or flat regions while increasing the number of epochs required for convergence, whereas an excessively large learning rate risks inducing oscillations or even divergence during training. Therefore, this study opts to start with a relatively small initial learning rate and gradually increase its value to identify the optimal setting. Figure 10 presents the measurement results of models configured with different initial learning rates, while Table 5 lists their corresponding performance metrics.

As the initial learning rate gradually increases from the minimum tested value, the model’s test performance improves accordingly. However, when the initial learning rate reaches $1\times 10^{-3}$, the model performance instead exhibits a decline. This may be attributed to the excessively large initial learning rate exceeding the effective adjustment range of the Adam optimizer, preventing the model from converging to optimal parameters. Therefore, the models discussed in this paper adopt an initial learning rate of $5\times 10^{-4}$.

**Figure 11:**
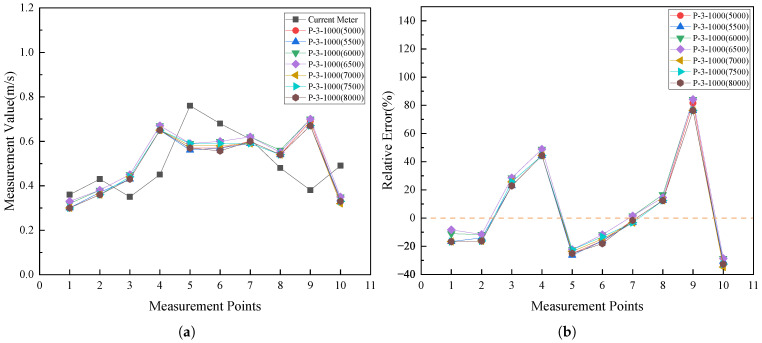
Results of epoch tests. (**a**) Measured values of different models; (**b**) relative errors of different models, with the zero-error line represented by a dashed line.

In the training epoch experiments, we commenced with 5000 epochs and progressively increased the iteration count to identify the optimal configuration. The measurement results for models trained under different epoch settings are presented in Figure 11, with the corresponding performance metrics summarized in Table 6.

In the epoch experiments, the model performance exhibited an overall upward trend as the number of training epochs increased. However, when the training epochs reached 8000, the performance showed a noticeable decline instead. This may be attributed to the excessively long training process causing the model to overfit the training data, thereby undermining its adaptability to diverse scenarios. Therefore, 7500 is set as the number of training epochs for the models discussed in this paper.

#### 4.1.3. Method Comparison

**Figure 12:**
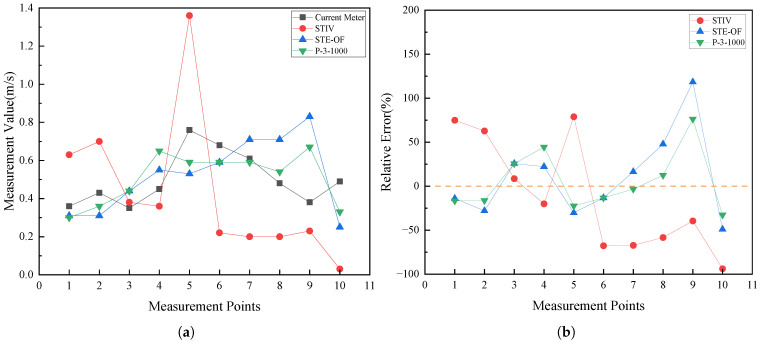
Measurement results of different methods at Dali Station. (**a**) Measured values of different methods; (**b**) relative errors of different methods, with the zero-error line represented by a dashed line.

Table 7 lists the measurement results of the current meter, the model with determined parameters, and the existing image-based flow measurement methods STIV [4] and STE-OF [35] at the Dali Hydrological Station. Figure 12 plots the flow velocities measured by different methods and the distribution of relative errors.

From the measurement results of the vertical average velocity presented in Table 7 and Figure 12, the proposed method performs satisfactorily in the regions near the camera and the river center. In these regions, the relative measurement errors at most velocity measurement points remain stable at approximately 20%. The most prominent relative error occurs at velocity measurement Point 9, with an error value reaching 76.32%, which indicates that the performance of the network model varies noticeably across different positions. This measuring point lies in close proximity to the riverbank, where severe light reflection interference is present in the captured images. Such optical distortion inevitably deteriorates the local flow velocity measurement accuracy. In addition, this monitoring point is relatively far from the camera perspective, which results in insufficient pixel definition and blurred imaging details in the corresponding local image area. This factor further aggravates and amplifies the measurement error at this location.

**Figure 13:**
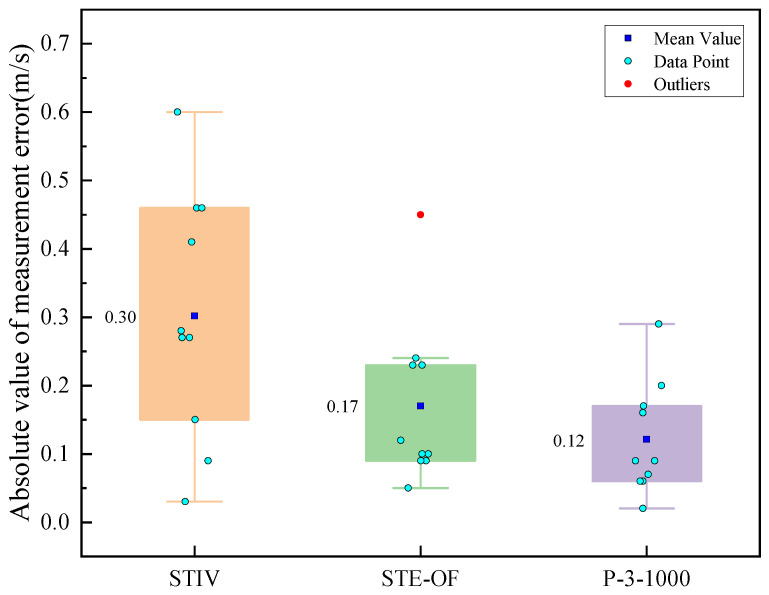
Absolute values of measured errors by different methods at Dali Station.

Figure 13 shows the distribution of absolute values of measurement errors obtained by different methods. Overall, the proposed method effectively reduces the measurement errors at all velocity measurement points. Compared with other methods, the errors generated by the proposed method are more closely distributed around zero, and no outliers appear, which indicates that the proposed method has better stability and higher accuracy.

This is also reflected in Table 8. The proposed method exhibits the lowest RMSE, and simultaneously, its SD is also the lowest. This indicates that the proposed method outperforms the other methods in overall measurement accuracy and robustness. In addition, flow velocities at most measurement points along both riverbanks are generally low, and the measured values at these locations tend to suffer from large deviations. Such discrepancies can significantly impair the overall correlation of the measurement results. Nevertheless, the Pearson correlation coefficient between the measured values of the proposed method and those of the current meter remains the highest. Compared with other measurement methods, the measurement results of the proposed method show a stronger correlation.

As shown in Table 7 and Figure 12, all the aforementioned methods exhibit certain errors at individual velocity measurement points compared to the current meter measurements. However, overall, the deviations of the total discharge and mean flow velocity obtained by these methods from the current meter measurements are controlled within a relatively small range.

In the process of calculating discharge using the velocity-area method, the total discharge can be regarded as a weighted sum of the velocity measurements, with the weights being the areas of the corresponding partial cross-sections. Consequently, the measurement errors at individual velocity measurement points are diluted during the summation process, which drives the final measurement results toward the true values.

Generally, the flow regime in the central region of a river is relatively stable with uniform illumination conditions, resulting in smaller measurement errors in this area. In contrast, regions near both banks are affected by factors such as camera perspective and flow conditions, leading to larger measurement errors. However, the partial cross-sectional areas in the central region are typically larger, which gives higher weights to the velocity measurements in this region. This weight distribution mechanism ensures that measurement values with large weights correspond to small errors, limiting their impact on the overall error. Although the measurement errors near the riverbanks are relatively large, their influence is diluted due to their smaller weights, ultimately maintaining the stability of the total flow field measurements.

On this basis, for the measurement of the overall flow field data, the total discharge calculated by the algorithm proposed in this paper is 4.59 $m^{3}/s$, and the average velocity is 0.473 m/s. Compared with the measurements from the current meter, the relative error of the total discharge is 0.66%, and the relative error of the average velocity is 0.64%, which are the smallest among all tested methods.

Preliminary experimental results indicate that in artificial river channel scenarios, the proposed method yields measurement results with smaller errors and higher correlation compared with the existing tested methods. Overall, this method allows for effective monitoring of river flow velocity and discharge, and exhibits favorable performance in the measurement experiments conducted in artificial river channels.

### 4.2. Results of Case Study 2

**Figure 14:**
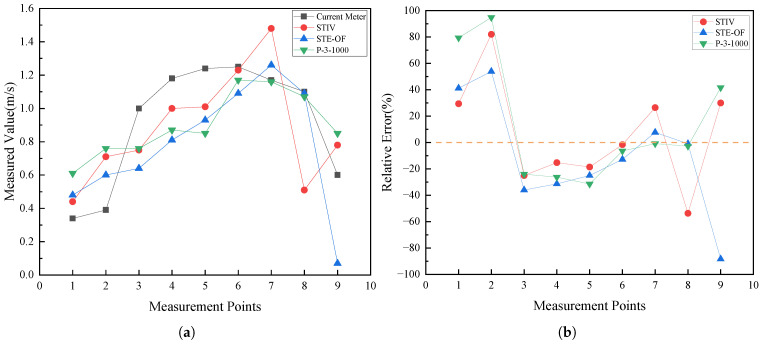
Measurement results of different methods at Chuxiong Station. (**a**) Measured values of different methods; (**b**) relative errors of different methods, with the zero-error line represented by a dashed line.

To verify that the tested model can generalize well to different rivers rather than being confined to a specific scenario, the model employed in Case 2 is kept consistent with that in Case 1, consisting of three hidden layers with 1000 neurons in each. The measurement results of the parameter-determined model are compared with those of the conventional STIV and STE-OF methods. The measurement results of different methods are shown in Table 9 and Figure 14.

Analysis of the data in Table 9 and Figure 14 shows that, in terms of measurement accuracy at different velocity measurement points, the proposed method yields relatively small errors in the central region of the river, which is consistent with the results of Case 1. In particular, high-precision measurement results are obtained by this method at velocity measurement points 6, 7 and 8, with all relative errors below 10%. In contrast, comparatively large measurement errors are concentrated near both riverbanks, especially at velocity measurement points 1, 2, and 9, where the relative errors reach 79.41%, 94.87%, and 41.67%, respectively. Such spatial discrepancy can be attributed to a combination of multiple influencing factors. In near-bank regions, the hydrodynamic characteristics of the flow are strongly affected by the uneven topographic undulations of the riverbed, which further induce obvious variations in the local flow regime. Moreover, the water depth along the river banks is generally shallow. The frictional interaction between the surface flow and river beaches complicates the vertical distribution of flow layers, thereby increasing the difficulty of accurate velocity estimation in these near-bank areas. Furthermore, measurement points 1 and 2 on the right river bank suffer from severe perspective distortion, which impedes the reliable extraction and accurate characterization of river flow motion features. In addition, point 9 near the left river bank is far from the camera, so the measurement accuracy is also limited by the clarity of the captured images.

**Figure 15:**
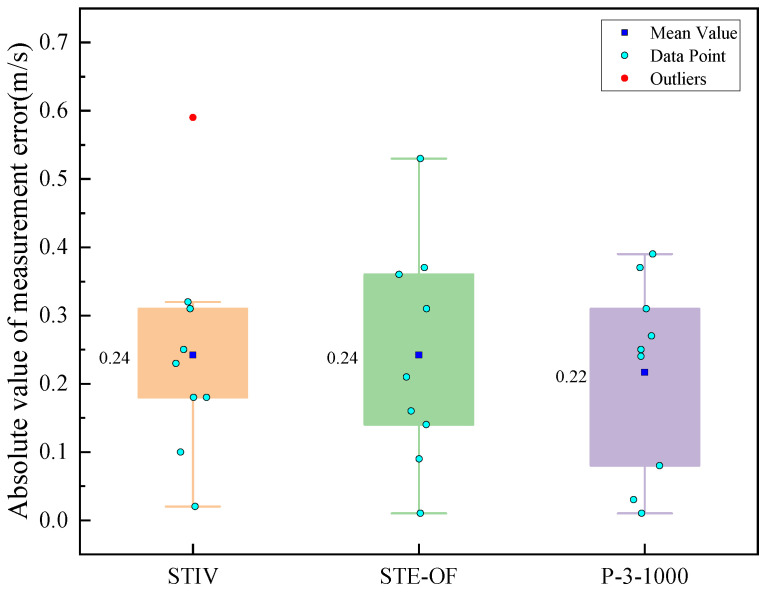
Absolute values of measured errors by different methods at Chuxiong Station.

Figure 15 presents a statistical analysis of the measurement errors of different methods. It can be seen that the minimum absolute measurement errors of the three methods are similar, while the overall distribution of the measurement errors of the STIV and STE-OF methods is relatively high. In contrast, the average error obtained by the proposed method is closer to 0, and the occurrence of local outliers is avoided.

In addition, the performance indicators in Table 10 demonstrate that among the tested methods, the proposed method achieves the smallest RMSE, which indicates that this method has the highest overall accuracy among the compared approaches. Meanwhile, the smallest SD also indicates that it possesses better measurement robustness compared to the other methods. In terms of the Pearson correlation coefficient, although the value of the proposed method is lower than that of the STE-OF method, it still shows a stronger correlation compared with the STIV method.

Meanwhile, the data in Table 9 shows that compared with the true values measured by the current meter, the relative error of total discharge obtained by the proposed method is −1.75%, the absolute value of which is smaller than −3.45% of the STIV method and −12.34% of the STE-OF method. The relative error of average flow velocity is −2.33%, the absolute value of which is also smaller than −3.49% of the STIV method and −12.79% of the STE-OF method. The proposed method therefore presents results closer to those measured by the current meter.

These results indicate that in natural river environments with restricted shooting conditions, the proposed method can still obtain measurement results closer to those measured by the current meter relative to the existing STIV and STE-OF methods, and achieve higher-precision measurement of river flow velocity and discharge.

## 5. Discussion

Image-based flow measurement algorithms such as STIV and optical flow have been widely applied to river surface velocity measurement due to their simplicity and non-intrusive nature. With the advancement of deep learning technology, the introduction of neural network models has also brought new momentum to the development of these methods. However, all these methods measure flow velocity by capturing and processing pixel variations in images. The shortage of physical knowledge makes it difficult for them to characterize the complex physical properties of water flow, leading to unsatisfactory measurement accuracy. Meanwhile, the lack of river datasets brings challenges to the training of deep learning methods that highly depend on labeled data. The inherent black-box characteristic of traditional neural network models also reduces the credibility of model decisions. On this basis, the proposed flow measurement algorithm integrated with optical flow and unlabeled PINN provides a new way to identify river surface velocity. Compared with existing methods, this method enhances the accuracy of river flow velocity and discharge estimation and offers certain physical support for the measurement results. Nevertheless, all river flow velocity and discharge measurement methods have their respective merits and limitations.

Environmental factors such as illumination may affect the performance of the algorithm. Compared with the STIV method that measures flow velocity along a single velocity line, the proposed method calculates the river flow velocity at individual velocity measurement points and may be more susceptible to environmental noise. Since explicit image processing methods (e.g., Gaussian filtering, histogram equalization) alter the distribution of pixel gradients, which violates the proportionality assumption between image grayscale and actual concentration, this study is conducted directly on images after projection transformation and grayscale conversion. To enhance the robustness of the method against noise, the loss function is set to MAE, complemented by a multi-scenario training strategy. The experimental scenarios cover typical operational conditions for river monitoring, encompassing both controlled artificial channels with regular geometries and stable flows, and natural rivers with irregular boundaries, variable flow regimes, and moderate perspective distortions. While these scenarios represent common field conditions encountered in hydrometric practice, the applicability of the proposed method under more extreme environmental conditions—such as intense illumination variations, heavy sediment loads, or nighttime monitoring—remains to be fully validated. Future research will focus on developing noise-resistant preprocessing methods that preserve the grayscale-concentration correspondence, as well as conducting comprehensive tests in challenging environments to further expand the operational envelope of the algorithm.

Meanwhile, the measurement accuracy of a single velocity measurement point remains a critical indicator for all flow measurement methods based on the velocity-area principle. Although the proposed method achieves a smaller RMSE and exhibits better overall accuracy compared with traditional methods, lens distortion gives rise to large projection transformation errors near the two river banks, resulting in poor performance of the method at the velocity measurement points in these regions. In addition, the measurement of distances between field calibration points and the selection of calibration points on captured images can also give rise to measurement errors during the projection transformation process. Therefore, follow-up research will focus on standardizing the projection transformation procedure to reduce the adverse impacts of such issues on measurement accuracy.

In addition, it should be noted that the method proposed in this paper is methodologically different from the existing deep learning methods for river flow velocity measurement. Traditional deep learning methods usually require a large amount of labeled training data, and are typically trained on public datasets before being transferred to the river scenarios for testing. Most existing PINN-based methods need to obtain labeled data from target scenarios for training, which limits their cross-scenario application capability. In contrast, the method proposed in this paper adopts an unlabeled training form, which is an important difference from existing methods. Direct comparative testing with supervised architectures makes it difficult to distinguish whether performance differences stem from the model architecture itself or the differences in training data. Therefore, how to carry out comparisons of deep learning methods with different paradigms under rigorous conditions is also an important direction to be explored in subsequent research.

## 6. Conclusions

To address the problems of traditional image-based flow measurement algorithms lacking a physical basis and having low accuracy in estimating river surface velocity and discharge, in this paper, an estimation method using optical flow and physics-informed neural networks has been proposed. This method introduces the convection–diffusion equation on the basis of optical flow and uses deep learning methods to solve and analyze flow velocity. While improving measurement accuracy, it provides certain physical interpretability for the obtained results, which is more consistent with the time-varying motion laws of rivers.

As an unlabeled data-driven method, this study eliminates the dependence of traditional supervised learning on the labeling of true flow velocity values, which is achieved by incorporating fluid mechanics equations into the loss function and introducing multi-scenario data. Unlike standard PINNs that take spatiotemporal coordinates as inputs and calculate partial derivatives through automatic differentiation, due to the discrete nature of image data, the method proposed in this paper uses gray gradients calculated by the finite difference scheme as the input features of the network, directly establishing a mapping relationship between pixel gradients and flow velocity. Meanwhile, to enable the model to avoid the limitations of traditional PINNs to a certain extent, the constraints of boundary conditions and initial conditions on the model are omitted from the loss function. Instead, data collected from different river scenarios are used to train the model to enhance its generalization ability.

Two test cases are employed to evaluate the performance of the model, and the effectiveness of the proposed method is verified through experiments in the artificial river channel and the natural river environment. In experiments under both scenarios, the measurement method presented in this paper achieves the smallest RMSE and exhibits good correlation with the measurement results of the current meter. Meanwhile, in the measurement of total discharge and average flow velocity, the proposed method is closer to the values measured by the current meter and realizes effective monitoring of river surface flow velocity and discharge.

Compared with traditional algorithms, the method proposed in this paper improves the accuracy of river flow velocity and discharge measurement to a certain extent, demonstrating its application potential in hydrometric measurements. Future research will focus on optimizing the performance of the algorithm under various environmental conditions and reducing the influence of lens distortion on measurement results, aiming to achieve high-precision estimation of river flow velocity and discharge in complex environments.

## Figures and Tables

**Table 1:** Performance comparison of initial tests.

Model	RMSE
P-1-100	0.2058
P-1-200	0.1771
P-2-100	0.1489

**Table 2:** Performance comparison of network layer-number tests.

Model	RMSE
P-1-100	0.2058
P-2-100	0.1489
P-3-100	0.1417
P-4-100	0.1625

**Table 3:** Performance comparison of neuron-number tests.

Model	RMSE	Pearson Coefficient
P-3-100	0.1417	0.3326
P-3-200	0.1494	0.3372
P-3-500	0.1464	0.3685
P-3-1000	0.1440	0.4016
P-3-1500	0.1493	0.3803

**Table 4:** Performance comparison of activation function tests.

Activation Function	RMSE	Pearson Coefficient
ReLU	0.1440	0.4016
Sigmoid	0.1478	0.4031
Tanh	0.1459	0.3360

**Table 5:** Performance comparison of learning rate tests.

Learning Rate	RMSE	Pearson Coefficient
$5\times 10^{-5}$	0.1506	0.2994
$1\times 10^{-4}$	0.1484	0.3632
$5\times 10^{-4}$	0.1440	0.4016
$1\times 10^{-3}$	0.1523	0.2336

**Table 6:** Performance comparison of epoch tests.

Epoch	RMSE	Pearson Coefficient
5000	0.1517	0.3402
5500	0.1471	0.3522
6000	0.1504	0.3669
6500	0.1497	0.3649
7000	0.1470	0.3761
7500	0.1440	0.4016
8000	0.1471	0.3686

**Table 7:** Comparison of measured flow velocity and discharge by different methods at Dali Station.

Measurement Point	Measured Value by Current Meter (m/s)	STIV		STE-OF		P-3-1000	
		Measured Value (m/s)	Relative Error (%)	Measured Value (m/s)	Relative Error (%)	Measured Value (m/s)	Relative Error (%)
1	0.36	0.63	75.00	0.31	−13.89	0.30	−16.67
2	0.43	0.70	62.79	0.31	−27.91	0.36	−16.28
3	0.35	0.38	8.57	0.44	25.71	0.44	25.71
4	0.45	0.36	−20.00	0.55	22.22	0.65	44.44
5	0.76	1.36	78.95	0.53	−30.26	0.59	−22.37
6	0.68	0.22	−67.65	0.59	−13.24	0.59	−13.24
7	0.61	0.20	−67.21	0.71	16.39	0.59	−3.28
8	0.48	0.20	−58.33	0.71	47.92	0.54	12.50
9	0.38	0.23	−39.47	0.83	118.42	0.67	76.32
10	0.49	0.03	−93.88	0.25	−48.98	0.33	−32.65
Total Discharge($m^{3}/s$)	4.56	4.36	−4.39	4.71	3.29	4.59	0.66
Average Velocity(m/s)	0.47	0.45	−4.26	0.49	4.26	0.47 *	0.64

* unrounded value: 0.473 m/s. Relative error (0.64%) computed from unrounded value to avoid misleading zero-error appearance.

**Table 8:** Performance comparison of different methods at Dali Station.

Method	RMSE	SD	Pearson Coefficient
STIV	0.3474	0.1810	0.3614
STE-OF	0.2047	0.1202	0.2135
P-3-1000	0.1440	0.0823	0.4016

**Table 9:** Comparison of measured flow velocity and discharge by different methods at Chuxiong Station.

Measurement Point	Measured Value by Current Meter (m/s)	STIV		STE-OF		P-3-1000	
		Measured Value (m/s)	Relative Error (%)	Measured Value (m/s)	Relative Error (%)	Measured Value (m/s)	Relative Error (%)
1	0.34	0.44	29.41	0.48	41.18	0.61	79.41
2	0.39	0.71	82.05	0.60	53.85	0.76	94.87
3	1.00	0.75	−25.00	0.64	−36.00	0.76	−24.00
4	1.18	1.00	−15.25	0.81	−31.36	0.87	−26.27
5	1.24	1.01	−18.55	0.93	−25.00	0.85	−31.45
6	1.25	1.23	−1.60	1.09	−12.80	1.17	−6.40
7	1.17	1.48	26.50	1.26	7.69	1.16	−0.85
8	1.10	0.51	−53.64	1.09	−0.91	1.07	−2.73
9	0.60	0.78	30.00	0.07	−88.33	0.85	41.67
Total Discharge($m^{3}/s$)	66.72	64.42	−3.45	58.49	−12.34	65.55	−1.75
Average Velocity(m/s)	0.86	0.83	−3.49	0.75	−12.79	0.84	−2.33

**Table 10:** Performance comparison of different methods at Chuxiong Station.

Method	RMSE	SD	Pearson Coefficient
STIV	0.2862	0.1617	0.6418
STE-OF	0.2869	0.1632	0.7476
P-3-1000	0.2551	0.1427	0.7121

## Data Availability

The data supporting the findings of this study can be found within the article.
